# Supplementary material for: Psychosocial supports within pediatric nephrology practices: A pediatric nephrology research consortium survey
Source: PLoS One. 2023 May 9;18(5):e0285126. doi: 10.1371/journal.pone.0285126 (PMC10168552; doi:10.1371/journal.pone.0285126)
Supplement: Attachment — Submitted filename: Dawson Response to Reviewers Letter 3.14.23.docx [file pone.0285126.s002.docx]

3/31/2023

**RE: Manuscript ID PONE-D-22-323765**

Dear Dr. Elrggal,

Thank you for your action letter received on 14 February 2023, which included the critiques from two reviewers for the manuscript, *Psychosocial Supports within Pediatric Nephrology Practices: A Pediatric Nephrology Research Consortium Survey.* My co-authors and I appreciate the reviews and the opportunity to revise this manuscript for resubmission.

Below, please find our point-by-point responses, with the quote of each reviewer followed by our response. We have also attempted to highlight the substantive changes made within the body of the resubmitted document. We appreciate the encouraging comments from the reviewers that our manuscript was well conducted, written, and will help contribute to change in pediatric nephrology practices. We are thankful for the recommendations we received, and we hope you agree that this manuscript is now a stronger report.

| **Journal Requirements** | |
| --- | --- |
| Reviewer comment | Revise and resubmit response |
| 1. Please ensure that your manuscript meets PLOS ONE's style requirements, including those for file naming. The PLOS ONE style templates can be found at  [https://journals.plos.org/plosone/s/file?id=wjVg/PLOSOne_formatting_sample_main_body.pdf](https://urldefense.com/v3/__https:/nam12.safelinks.protection.outlook.com/?url=https*3A*2F*2Furldefense.com*2Fv3*2F__https*3A*2Fjournals.plos.org*2Fplosone*2Fs*2Ffile*3Fid*3DwjVg*PLOSOne_formatting_sample_main_body.pdf__*3BLw!!NiUAmZJ8c1GNWg!TBonR0TS8UgvchhDKCN7EPhRcjzHOXxHUihN9TWC-rbJqbnruXC_nEBCZ1bKY-kTpWJbXhzd-ogvIZaOS44eUYY4oyk*24&data=05*7C01*7Cjulamott*40iu.edu*7C12213440a5884b748ddb08db0fa72791*7C1113be34aed14d00ab4bcdd02510be91*7C0*7C0*7C638120985055783396*7CUnknown*7CTWFpbGZsb3d8eyJWIjoiMC4wLjAwMDAiLCJQIjoiV2luMzIiLCJBTiI6Ik1haWwiLCJXVCI6Mn0*3D*7C3000*7C*7C*7C&sdata=g94puerkp0FT*2FqKmzyx4YisF0rX3uE0u6ayOcUtQ*2Fso*3D&reserved=0__;JSUlJSUlJSUlJSUlKiUlJSUlJSUlJSUlJSUlJSUlJSUl!!NiUAmZJ8c1GNWg!R1eFraNmbSbbW1nebu0-MW9M2DoV0-DY0w9gq1e-N7bSduYSWLQ3sBWMxmrCFuTDeJvTGAQkzp7n3R2lDQ24i3XfivVTZ1Y$) and  [https://journals.plos.org/plosone/s/file?id=ba62/PLOSOne_formatting_sample_title_authors_affiliations.pdf](https://urldefense.com/v3/__https:/nam12.safelinks.protection.outlook.com/?url=https*3A*2F*2Furldefense.com*2Fv3*2F__https*3A*2Fjournals.plos.org*2Fplosone*2Fs*2Ffile*3Fid*3Dba62*PLOSOne_formatting_sample_title_authors_affiliations.pdf__*3BLw!!NiUAmZJ8c1GNWg!TBonR0TS8UgvchhDKCN7EPhRcjzHOXxHUihN9TWC-rbJqbnruXC_nEBCZ1bKY-kTpWJbXhzd-ogvIZaOS44eEJDHOsA*24&data=05*7C01*7Cjulamott*40iu.edu*7C12213440a5884b748ddb08db0fa72791*7C1113be34aed14d00ab4bcdd02510be91*7C0*7C0*7C638120985055783396*7CUnknown*7CTWFpbGZsb3d8eyJWIjoiMC4wLjAwMDAiLCJQIjoiV2luMzIiLCJBTiI6Ik1haWwiLCJXVCI6Mn0*3D*7C3000*7C*7C*7C&sdata=ryE0newHq97hmGJHsR3mdop2VLlcouGgRguXJkWivvo*3D&reserved=0__;JSUlJSUlJSUlJSUlKiUlJSUlJSUlJSUlJSUlJSUlJQ!!NiUAmZJ8c1GNWg!R1eFraNmbSbbW1nebu0-MW9M2DoV0-DY0w9gq1e-N7bSduYSWLQ3sBWMxmrCFuTDeJvTGAQkzp7n3R2lDQ24i3XfGKw0Ahc$) | We referenced the style templates and adjusted our headings, figure/tables, and citations according to the template guide. |
| 2. Please provide additional details regarding participant consent. In the ethics statement in the Methods and online submission information, please ensure that you have specified what type you obtained (for instance, written or verbal, and if verbal, how it was documented and witnessed). If your study included minors, state whether you obtained consent from parents or guardians. If the need for consent was waived by the ethics committee, please include this information. | Thank you for the recommendation to outline our consent process in greater detail. We have added text to the document that addresses the areas requested in our Methods, specifically that our research was determined by our institutions as not human subjects and consent was waived. |
| 3. Please include your full ethics statement in the ‘Methods’ section of your manuscript file. In your statement, please include the full name of the IRB or ethics committee who approved or waived your study, as well as whether or not you obtained informed written or verbal consent. If consent was waived for your study, please include this information in your statement as well. | Thank you for the request to make more specific the ethics statement in our manuscript’s Methods section. As stated above, we have included more specific language that outlines the waiver that was granted by both institutions sponsoring the project. |
| 4. We note that Figure 1 in your submission contain [map/satellite] images which may be copyrighted. All PLOS content is published under the Creative Commons Attribution License (CC BY 4.0), which means that the manuscript, images, and Supporting Information files will be freely available online, and any third party is permitted to access, download, copy, distribute, and use these materials in any way, even commercially, with proper attribution. For these reasons, we cannot publish previously copyrighted maps or satellite images created using proprietary data, such as Google software (Google Maps, Street View, and Earth). For more information, see our copyright guidelines: [http://journals.plos.org/plosone/s/licenses-and-copyright](https://urldefense.com/v3/__https:/nam12.safelinks.protection.outlook.com/?url=https*3A*2F*2Furldefense.com*2Fv3*2F__http*3A*2Fjournals.plos.org*2Fplosone*2Fs*2Flicenses-and-copyright__*3B!!NiUAmZJ8c1GNWg!TBonR0TS8UgvchhDKCN7EPhRcjzHOXxHUihN9TWC-rbJqbnruXC_nEBCZ1bKY-kTpWJbXhzd-ogvIZaOS44epVT2HWk*24&data=05*7C01*7Cjulamott*40iu.edu*7C12213440a5884b748ddb08db0fa72791*7C1113be34aed14d00ab4bcdd02510be91*7C0*7C0*7C638120985055783396*7CUnknown*7CTWFpbGZsb3d8eyJWIjoiMC4wLjAwMDAiLCJQIjoiV2luMzIiLCJBTiI6Ik1haWwiLCJXVCI6Mn0*3D*7C3000*7C*7C*7C&sdata=uvQ*2B2YsuZUdyK1XCeLOmUjk*2FuDH3fPgkZ9Yb8EzgaSU*3D&reserved=0__;JSUlJSUlJSUlJSUlJSUlJSUlJSUlJSUlJSUlJSUl!!NiUAmZJ8c1GNWg!R1eFraNmbSbbW1nebu0-MW9M2DoV0-DY0w9gq1e-N7bSduYSWLQ3sBWMxmrCFuTDeJvTGAQkzp7n3R2lDQ24i3XfA_69muQ$).  We require you to either (1) present written permission from the copyright holder to publish these figures specifically under the CC BY 4.0 license, or (2) remove the figures from your submission:     a. You may seek permission from the original copyright holder of Figure(s) [#] to publish the content specifically under the CC BY 4.0 license.  We recommend that you contact the original copyright holder with the Content Permission Form ([http://journals.plos.org/plosone/s/file?id=7c09/content-permission-form.pdf](https://urldefense.com/v3/__https:/nam12.safelinks.protection.outlook.com/?url=https*3A*2F*2Furldefense.com*2Fv3*2F__http*3A*2Fjournals.plos.org*2Fplosone*2Fs*2Ffile*3Fid*3D7c09*content-permission-form.pdf__*3BLw!!NiUAmZJ8c1GNWg!TBonR0TS8UgvchhDKCN7EPhRcjzHOXxHUihN9TWC-rbJqbnruXC_nEBCZ1bKY-kTpWJbXhzd-ogvIZaOS44ehwPjVvs*24&data=05*7C01*7Cjulamott*40iu.edu*7C12213440a5884b748ddb08db0fa72791*7C1113be34aed14d00ab4bcdd02510be91*7C0*7C0*7C638120985055783396*7CUnknown*7CTWFpbGZsb3d8eyJWIjoiMC4wLjAwMDAiLCJQIjoiV2luMzIiLCJBTiI6Ik1haWwiLCJXVCI6Mn0*3D*7C3000*7C*7C*7C&sdata=x9*2FHx7oej33XMJbknJ9r02whsR8fStz7EEAM8TLClwA*3D&reserved=0__;JSUlJSUlJSUlJSUlKiUlJSUlJSUlJSUlJSUlJSUlJSU!!NiUAmZJ8c1GNWg!R1eFraNmbSbbW1nebu0-MW9M2DoV0-DY0w9gq1e-N7bSduYSWLQ3sBWMxmrCFuTDeJvTGAQkzp7n3R2lDQ24i3XfZz6jpAI$)) and the following text:  “I request permission for the open-access journal PLOS ONE to publish XXX under the Creative Commons Attribution License (CCAL) CC BY 4.0 ([http://creativecommons.org/licenses/by/4.0/](https://urldefense.com/v3/__https:/nam12.safelinks.protection.outlook.com/?url=https*3A*2F*2Furldefense.com*2Fv3*2F__http*3A*2Fcreativecommons.org*2Flicenses*2Fby*2F4.0*2F__*3B!!NiUAmZJ8c1GNWg!TBonR0TS8UgvchhDKCN7EPhRcjzHOXxHUihN9TWC-rbJqbnruXC_nEBCZ1bKY-kTpWJbXhzd-ogvIZaOS44ei-kQzko*24&data=05*7C01*7Cjulamott*40iu.edu*7C12213440a5884b748ddb08db0fa72791*7C1113be34aed14d00ab4bcdd02510be91*7C0*7C0*7C638120985055939607*7CUnknown*7CTWFpbGZsb3d8eyJWIjoiMC4wLjAwMDAiLCJQIjoiV2luMzIiLCJBTiI6Ik1haWwiLCJXVCI6Mn0*3D*7C3000*7C*7C*7C&sdata=aanQZ3ErCXBpqxBXsXmXG2Mh5B*2BQxFm6AHSey7l9tpc*3D&reserved=0__;JSUlJSUlJSUlJSUlJSUlJSUlJSUlJSUlJSUlJSUl!!NiUAmZJ8c1GNWg!R1eFraNmbSbbW1nebu0-MW9M2DoV0-DY0w9gq1e-N7bSduYSWLQ3sBWMxmrCFuTDeJvTGAQkzp7n3R2lDQ24i3XfS1PI9z8$)). Please be aware that this license allows unrestricted use and distribution, even commercially, by third parties. Please reply and provide explicit written permission to publish XXX under a CC BY license and complete the attached form.”  Please upload the completed Content Permission Form or other proof of granted permissions as an "Other" file with your submission.  In the figure caption of the copyrighted figure, please include the following text: “Reprinted from [ref] under a CC BY license, with permission from [name of publisher], original copyright [original copyright year].”       b. If you are unable to obtain permission from the original copyright holder to publish these figures under the CC BY 4.0 license or if the copyright holder’s requirements are incompatible with the CC BY 4.0 license, please either i) remove the figure or ii) supply a replacement figure that complies with the CC BY 4.0 license. Please check copyright information on all replacement figures and update the figure caption with source information. If applicable, please specify in the figure caption text when a figure is similar but not identical to the original image and is therefore for illustrative purposes only.  The following resources for replacing copyrighted map figures may be helpful:   USGS National Map Viewer (public domain): [http://viewer.nationalmap.gov/viewer/](https://urldefense.com/v3/__https:/nam12.safelinks.protection.outlook.com/?url=https*3A*2F*2Furldefense.com*2Fv3*2F__http*3A*2Fviewer.nationalmap.gov*2Fviewer*2F__*3B!!NiUAmZJ8c1GNWg!TBonR0TS8UgvchhDKCN7EPhRcjzHOXxHUihN9TWC-rbJqbnruXC_nEBCZ1bKY-kTpWJbXhzd-ogvIZaOS44eoTYD04Y*24&data=05*7C01*7Cjulamott*40iu.edu*7C12213440a5884b748ddb08db0fa72791*7C1113be34aed14d00ab4bcdd02510be91*7C0*7C0*7C638120985055939607*7CUnknown*7CTWFpbGZsb3d8eyJWIjoiMC4wLjAwMDAiLCJQIjoiV2luMzIiLCJBTiI6Ik1haWwiLCJXVCI6Mn0*3D*7C3000*7C*7C*7C&sdata=OA7gxIdFe72Ns4CA*2BdkJGSDV4CdeR7ymZanGgAzqPiI*3D&reserved=0__;JSUlJSUlJSUlJSUlJSUlJSUlJSUlJSUlJSUlJQ!!NiUAmZJ8c1GNWg!R1eFraNmbSbbW1nebu0-MW9M2DoV0-DY0w9gq1e-N7bSduYSWLQ3sBWMxmrCFuTDeJvTGAQkzp7n3R2lDQ24i3XfPUPKOhA$)  The Gateway to Astronaut Photography of Earth (public domain):  [http://eol.jsc.nasa.gov/sseop/clickmap/](https://urldefense.com/v3/__https:/nam12.safelinks.protection.outlook.com/?url=https*3A*2F*2Furldefense.com*2Fv3*2F__http*3A*2Feol.jsc.nasa.gov*2Fsseop*2Fclickmap*2F__*3B!!NiUAmZJ8c1GNWg!TBonR0TS8UgvchhDKCN7EPhRcjzHOXxHUihN9TWC-rbJqbnruXC_nEBCZ1bKY-kTpWJbXhzd-ogvIZaOS44ejYiplhE*24&data=05*7C01*7Cjulamott*40iu.edu*7C12213440a5884b748ddb08db0fa72791*7C1113be34aed14d00ab4bcdd02510be91*7C0*7C0*7C638120985055939607*7CUnknown*7CTWFpbGZsb3d8eyJWIjoiMC4wLjAwMDAiLCJQIjoiV2luMzIiLCJBTiI6Ik1haWwiLCJXVCI6Mn0*3D*7C3000*7C*7C*7C&sdata=clGHy3HGRzxkm0I790FABYfBF0gd2U6*2BrrdPmaoGpws*3D&reserved=0__;JSUlJSUlJSUlJSUlJSUlJSUlJSUlJSUlJSUlJSU!!NiUAmZJ8c1GNWg!R1eFraNmbSbbW1nebu0-MW9M2DoV0-DY0w9gq1e-N7bSduYSWLQ3sBWMxmrCFuTDeJvTGAQkzp7n3R2lDQ24i3XfTDv_Y7U$)  Maps at the CIA (public domain): [https://www.cia.gov/library/publications/the-world-factbook/index.html](https://urldefense.com/v3/__https:/nam12.safelinks.protection.outlook.com/?url=https*3A*2F*2Furldefense.com*2Fv3*2F__https*3A*2Fwww.cia.gov*2Flibrary*2Fpublications*2Fthe-world-factbook*2Findex.html__*3B!!NiUAmZJ8c1GNWg!TBonR0TS8UgvchhDKCN7EPhRcjzHOXxHUihN9TWC-rbJqbnruXC_nEBCZ1bKY-kTpWJbXhzd-ogvIZaOS44etRxsDKk*24&data=05*7C01*7Cjulamott*40iu.edu*7C12213440a5884b748ddb08db0fa72791*7C1113be34aed14d00ab4bcdd02510be91*7C0*7C0*7C638120985055939607*7CUnknown*7CTWFpbGZsb3d8eyJWIjoiMC4wLjAwMDAiLCJQIjoiV2luMzIiLCJBTiI6Ik1haWwiLCJXVCI6Mn0*3D*7C3000*7C*7C*7C&sdata=GIucOKSPCipAWXvVGob7dp4PkIZ5z0R2TGLygzCMRtY*3D&reserved=0__;JSUlJSUlJSUlJSUlJSUlJSUlJSUlJSUlJSUlJSU!!NiUAmZJ8c1GNWg!R1eFraNmbSbbW1nebu0-MW9M2DoV0-DY0w9gq1e-N7bSduYSWLQ3sBWMxmrCFuTDeJvTGAQkzp7n3R2lDQ24i3Xfr3IttYA$) and [https://www.cia.gov/library/publications/cia-maps-publications/index.html](https://urldefense.com/v3/__https:/nam12.safelinks.protection.outlook.com/?url=https*3A*2F*2Furldefense.com*2Fv3*2F__https*3A*2Fwww.cia.gov*2Flibrary*2Fpublications*2Fcia-maps-publications*2Findex.html__*3B!!NiUAmZJ8c1GNWg!TBonR0TS8UgvchhDKCN7EPhRcjzHOXxHUihN9TWC-rbJqbnruXC_nEBCZ1bKY-kTpWJbXhzd-ogvIZaOS44e9ceumeA*24&data=05*7C01*7Cjulamott*40iu.edu*7C12213440a5884b748ddb08db0fa72791*7C1113be34aed14d00ab4bcdd02510be91*7C0*7C0*7C638120985055939607*7CUnknown*7CTWFpbGZsb3d8eyJWIjoiMC4wLjAwMDAiLCJQIjoiV2luMzIiLCJBTiI6Ik1haWwiLCJXVCI6Mn0*3D*7C3000*7C*7C*7C&sdata=0orGpue6uXG9QjAcU6R5AxrKcWx7zGgPwKzAHsYOkUI*3D&reserved=0__;JSUlJSUlJSUlJSUlJSUlJSUlJSUlJSUlJSUlJSU!!NiUAmZJ8c1GNWg!R1eFraNmbSbbW1nebu0-MW9M2DoV0-DY0w9gq1e-N7bSduYSWLQ3sBWMxmrCFuTDeJvTGAQkzp7n3R2lDQ24i3XfE5kxn_U$)  NASA Earth Observatory (public domain): [http://earthobservatory.nasa.gov/](https://urldefense.com/v3/__https:/nam12.safelinks.protection.outlook.com/?url=https*3A*2F*2Furldefense.com*2Fv3*2F__http*3A*2Fearthobservatory.nasa.gov*2F__*3B!!NiUAmZJ8c1GNWg!TBonR0TS8UgvchhDKCN7EPhRcjzHOXxHUihN9TWC-rbJqbnruXC_nEBCZ1bKY-kTpWJbXhzd-ogvIZaOS44eawjwBac*24&data=05*7C01*7Cjulamott*40iu.edu*7C12213440a5884b748ddb08db0fa72791*7C1113be34aed14d00ab4bcdd02510be91*7C0*7C0*7C638120985055939607*7CUnknown*7CTWFpbGZsb3d8eyJWIjoiMC4wLjAwMDAiLCJQIjoiV2luMzIiLCJBTiI6Ik1haWwiLCJXVCI6Mn0*3D*7C3000*7C*7C*7C&sdata=kuOIAXG1HaZvmIsJjyPTxuYiiSqyOIyFAl7aTfCqf9U*3D&reserved=0__;JSUlJSUlJSUlJSUlJSUlJSUlJSUlJSUlJSU!!NiUAmZJ8c1GNWg!R1eFraNmbSbbW1nebu0-MW9M2DoV0-DY0w9gq1e-N7bSduYSWLQ3sBWMxmrCFuTDeJvTGAQkzp7n3R2lDQ24i3XfjNB-z2w$)  Landsat: [http://landsat.visibleearth.nasa.gov/](https://urldefense.com/v3/__https:/nam12.safelinks.protection.outlook.com/?url=https*3A*2F*2Furldefense.com*2Fv3*2F__http*3A*2Flandsat.visibleearth.nasa.gov*2F__*3B!!NiUAmZJ8c1GNWg!TBonR0TS8UgvchhDKCN7EPhRcjzHOXxHUihN9TWC-rbJqbnruXC_nEBCZ1bKY-kTpWJbXhzd-ogvIZaOS44eoWUwCPw*24&data=05*7C01*7Cjulamott*40iu.edu*7C12213440a5884b748ddb08db0fa72791*7C1113be34aed14d00ab4bcdd02510be91*7C0*7C0*7C638120985055939607*7CUnknown*7CTWFpbGZsb3d8eyJWIjoiMC4wLjAwMDAiLCJQIjoiV2luMzIiLCJBTiI6Ik1haWwiLCJXVCI6Mn0*3D*7C3000*7C*7C*7C&sdata=EjHV3jP7Nq3JBnKryWmY9F8pKwETchgeqNtp9aDzHe0*3D&reserved=0__;JSUlJSUlJSUlJSUlJSUlJSUlJSUlJSUlJSU!!NiUAmZJ8c1GNWg!R1eFraNmbSbbW1nebu0-MW9M2DoV0-DY0w9gq1e-N7bSduYSWLQ3sBWMxmrCFuTDeJvTGAQkzp7n3R2lDQ24i3XfjRG36xs$)  USGS EROS (Earth Resources Observatory and Science (EROS) Center) (public domain): [http://eros.usgs.gov/#](https://urldefense.com/v3/__https:/nam12.safelinks.protection.outlook.com/?url=https*3A*2F*2Furldefense.com*2Fv3*2F__http*3A*2Feros.usgs.gov*2F__*3B!!NiUAmZJ8c1GNWg!TBonR0TS8UgvchhDKCN7EPhRcjzHOXxHUihN9TWC-rbJqbnruXC_nEBCZ1bKY-kTpWJbXhzd-ogvIZaOS44eqYpZ7ao*24&data=05*7C01*7Cjulamott*40iu.edu*7C12213440a5884b748ddb08db0fa72791*7C1113be34aed14d00ab4bcdd02510be91*7C0*7C0*7C638120985055939607*7CUnknown*7CTWFpbGZsb3d8eyJWIjoiMC4wLjAwMDAiLCJQIjoiV2luMzIiLCJBTiI6Ik1haWwiLCJXVCI6Mn0*3D*7C3000*7C*7C*7C&sdata=tfkMj*2BGMvijpYDQD6hL0kscm*2BqwVaW0jf*2Bc2JmBJ1b4*3D&reserved=0__;JSUlJSUlJSUlJSUlJSUlJSUlJSUlJSUlJSUlJSU!!NiUAmZJ8c1GNWg!R1eFraNmbSbbW1nebu0-MW9M2DoV0-DY0w9gq1e-N7bSduYSWLQ3sBWMxmrCFuTDeJvTGAQkzp7n3R2lDQ24i3XfvVmPZAo$)  Natural Earth (public domain): [http://www.naturalearthdata.com/](https://urldefense.com/v3/__https:/nam12.safelinks.protection.outlook.com/?url=https*3A*2F*2Furldefense.com*2Fv3*2F__http*3A*2Fwww.naturalearthdata.com*2F__*3B!!NiUAmZJ8c1GNWg!TBonR0TS8UgvchhDKCN7EPhRcjzHOXxHUihN9TWC-rbJqbnruXC_nEBCZ1bKY-kTpWJbXhzd-ogvIZaOS44eqmCJR0I*24&data=05*7C01*7Cjulamott*40iu.edu*7C12213440a5884b748ddb08db0fa72791*7C1113be34aed14d00ab4bcdd02510be91*7C0*7C0*7C638120985055939607*7CUnknown*7CTWFpbGZsb3d8eyJWIjoiMC4wLjAwMDAiLCJQIjoiV2luMzIiLCJBTiI6Ik1haWwiLCJXVCI6Mn0*3D*7C3000*7C*7C*7C&sdata=vkn8RkEWPATxanv7eUmjZZYghgR*2FT1sfXKGJylh3S78*3D&reserved=0__;JSUlJSUlJSUlJSUlJSUlJSUlJSUlJSUlJSUl!!NiUAmZJ8c1GNWg!R1eFraNmbSbbW1nebu0-MW9M2DoV0-DY0w9gq1e-N7bSduYSWLQ3sBWMxmrCFuTDeJvTGAQkzp7n3R2lDQ24i3Xffj_UExI$) | Map images are in public domain searched through Google images. We switched to using a map that explicitly states the rights of use- for personal and commercial purposes, and do include reference to location map was found, per recommendation from the website: <https://simplemaps.com/resources/svg-license> |
| 5. In your Data Availability statement, you have not specified where the minimal data set underlying the results described in your manuscript can be found. PLOS defines a study's minimal data set as the underlying data used to reach the conclusions drawn in the manuscript and any additional data required to replicate the reported study findings in their entirety. All PLOS journals require that the minimal data set be made fully available. For more information about our data policy, please see [http://journals.plos.org/plosone/s/data-availability](https://urldefense.com/v3/__https:/nam12.safelinks.protection.outlook.com/?url=https*3A*2F*2Furldefense.com*2Fv3*2F__http*3A*2Fjournals.plos.org*2Fplosone*2Fs*2Fdata-availability__*3B!!NiUAmZJ8c1GNWg!TBonR0TS8UgvchhDKCN7EPhRcjzHOXxHUihN9TWC-rbJqbnruXC_nEBCZ1bKY-kTpWJbXhzd-ogvIZaOS44e67Geuus*24&data=05*7C01*7Cjulamott*40iu.edu*7C12213440a5884b748ddb08db0fa72791*7C1113be34aed14d00ab4bcdd02510be91*7C0*7C0*7C638120985055939607*7CUnknown*7CTWFpbGZsb3d8eyJWIjoiMC4wLjAwMDAiLCJQIjoiV2luMzIiLCJBTiI6Ik1haWwiLCJXVCI6Mn0*3D*7C3000*7C*7C*7C&sdata=0b0OLR*2BiNmnblptrISEYIjw1zwZ6PaLRJ0f*2F6GrinQg*3D&reserved=0__;JSUlJSUlJSUlJSUlJSUlJSUlJSUlJSUlJSUlJSUl!!NiUAmZJ8c1GNWg!R1eFraNmbSbbW1nebu0-MW9M2DoV0-DY0w9gq1e-N7bSduYSWLQ3sBWMxmrCFuTDeJvTGAQkzp7n3R2lDQ24i3Xf-0XHavs$).  Upon re-submitting your revised manuscript, please upload your study’s minimal underlying data set as either Supporting Information files or to a stable, public repository and include the relevant URLs, DOIs, or accession numbers within your revised cover letter. For a list of acceptable repositories, please see [http://journals.plos.org/plosone/s/data-availability#loc-recommended-repositories](https://urldefense.com/v3/__https:/nam12.safelinks.protection.outlook.com/?url=https*3A*2F*2Furldefense.com*2Fv3*2F__http*3A*2Fjournals.plos.org*2Fplosone*2Fs*2Fdata-availability*loc-recommended-repositories__*3BIw!!NiUAmZJ8c1GNWg!TBonR0TS8UgvchhDKCN7EPhRcjzHOXxHUihN9TWC-rbJqbnruXC_nEBCZ1bKY-kTpWJbXhzd-ogvIZaOS44ehJvAleM*24&data=05*7C01*7Cjulamott*40iu.edu*7C12213440a5884b748ddb08db0fa72791*7C1113be34aed14d00ab4bcdd02510be91*7C0*7C0*7C638120985055939607*7CUnknown*7CTWFpbGZsb3d8eyJWIjoiMC4wLjAwMDAiLCJQIjoiV2luMzIiLCJBTiI6Ik1haWwiLCJXVCI6Mn0*3D*7C3000*7C*7C*7C&sdata=4klHkPnNzIrEKWw0aez5mH4R*2BYjB7kcHJAiZv54AicU*3D&reserved=0__;JSUlJSUlJSUlJSolJSUlJSUlJSUlJSUlJSUlJSUl!!NiUAmZJ8c1GNWg!R1eFraNmbSbbW1nebu0-MW9M2DoV0-DY0w9gq1e-N7bSduYSWLQ3sBWMxmrCFuTDeJvTGAQkzp7n3R2lDQ24i3Xf6FG8ucw$). Any potentially identifying patient information must be fully anonymized.  Important: If there are ethical or legal restrictions to sharing your data publicly, please explain these restrictions in detail. Please see our guidelines for more information on what we consider unacceptable restrictions to publicly sharing data: [http://journals.plos.org/plosone/s/data-availability#loc-unacceptable-data-access-restrictions](https://urldefense.com/v3/__https:/nam12.safelinks.protection.outlook.com/?url=https*3A*2F*2Furldefense.com*2Fv3*2F__http*3A*2Fjournals.plos.org*2Fplosone*2Fs*2Fdata-availability*loc-unacceptable-data-access-restrictions__*3BIw!!NiUAmZJ8c1GNWg!TBonR0TS8UgvchhDKCN7EPhRcjzHOXxHUihN9TWC-rbJqbnruXC_nEBCZ1bKY-kTpWJbXhzd-ogvIZaOS44eA6iXj8w*24&data=05*7C01*7Cjulamott*40iu.edu*7C12213440a5884b748ddb08db0fa72791*7C1113be34aed14d00ab4bcdd02510be91*7C0*7C0*7C638120985055939607*7CUnknown*7CTWFpbGZsb3d8eyJWIjoiMC4wLjAwMDAiLCJQIjoiV2luMzIiLCJBTiI6Ik1haWwiLCJXVCI6Mn0*3D*7C3000*7C*7C*7C&sdata=BIpxxSxpZV1A*2FofJkgMbELhV8t*2B6FjAiUHd7oYB8CCQ*3D&reserved=0__;JSUlJSUlJSUlJSolJSUlJSUlJSUlJSUlJSUlJSUlJQ!!NiUAmZJ8c1GNWg!R1eFraNmbSbbW1nebu0-MW9M2DoV0-DY0w9gq1e-N7bSduYSWLQ3sBWMxmrCFuTDeJvTGAQkzp7n3R2lDQ24i3XfkNkYfA4$). Note that it is not acceptable for the authors to be the sole named individuals responsible for ensuring data access.  We will update your Data Availability statement to reflect the information you provide in your cover letter. | We are providing a copy of our dataset in SPSS format (i.e., .sav), though we have deleted the variables identifying PNRC site- as we indicated to participants that this information would not be shared. |
| 6. Please review your reference list to ensure that it is complete and correct. If you have cited papers that have been retracted, please include the rationale for doing so in the manuscript text, or remove these references and replace them with relevant current references. Any changes to the reference list should be mentioned in the rebuttal letter that accompanies your revised manuscript. If you need to cite a retracted article, indicate the article’s retracted status in the References list and also include a citation and full reference for the retraction notice. | Our reference list was reviewed and updated per additional citations included per recommendations of the reviewers. |
| **Reviewer 1** | |
| Thank you to Dawson et al for submitting this manuscript describing outcomes from a survey of paediatric nephrology centres across the USA in regards to psychosocial support services. It is well conducted and written. Thank you to the authors for the substantial work that has been undertaken. I only have minor queries.  1) It may be worth making mention of the diversity of stages and treatments of kidney disease amongst children as well as the etiologies, especially given that the burden of psychosocial support needs is likely different across the spectrum from early CKD to dialysis to transplant. | We appreciate the support for this manuscript. Related to comment 1, we briefly highlight the heterogeneity in the care required by pediatric nephrologists in the first paragraph of this report, and we have added supporting citations. |
| 2) Whilst all of the questions I had in regards to the PNRC are subsequently answered later in the methods, it would be better to include some of these early in the manuscript when the PNRC is first mentioned. | We added brief language to briefly introduce the purpose of the PNRC earlier in our introduction. |
| 3) More exploration of transition (ie, paediatric to adult services) in the context of psychosocial supports/services would be valued and increase the likely readership. | We wholeheartedly agree with the reviewer regarding the importance of the potential role of psychosocial providers to be involved in transition to adulthood needs for kidney patients and their families, and we have made reference to that in the discussion. Per your recommendation, we have also highlighted our additional sentence in the introduction calling attention to this important consideration. |
| 4) The discussion does feel quite long at times, but the content is highly valued. I would suggest a small table perhaps of the key take point points to help readers to engage with that content better. | Thank you for the suggestion of including a table of key take away points. We have included a summary of key findings/conclusions to help the reader see at a glance the main findings at the end of our discussion section. |
| **Reviewer 2** | |
| Reviewer #2: Thank you for this manuscribt which can make changing in ouur practice review the writing .there is spelling and grammar need revision. we need abbreviation of PNRC not clear more detailed.regarding discussion add reference at end of each paragraph | The Pediatric Nephrology Research Consortium is initially spelled out and briefly explained on pg. 4. Due to request to clarify the abbreviation, we again spell it out at the start of the Method section and Discussion section, when PNRC is re-introduced. We have also thoroughly reviewed our manuscript and corrected typos/grammars throughout where we could identify potential concerns. |
